# Normalized T1 Magnetic Resonance Imaging for Assessment of Regional Lung Function in Adult Cystic Fibrosis Patients - A Cross-Sectional Study

**DOI:** 10.1371/journal.pone.0073286

**Published:** 2013-09-25

**Authors:** Elliott C. Dasenbrook, Lan Lu, Shannon Donnola, David E. Weaver, Vikas Gulani, Peter M. Jakob, Michael W. Konstan, Chris A. Flask

**Affiliations:** 1 Department of Pediatrics, Case Western Reserve University School of Medicine, University Hospitals Case Medical Center and Rainbow Babies and Children's Hospital, Cleveland, Ohio, United States of America; 2 Department of Medicine, Case Western Reserve University School of Medicine, University Hospitals Case Medical Center and Rainbow Babies and Children's Hospital, Cleveland, Ohio, United States of America; 3 Department of Radiology, Case Western Reserve University School of Medicine, University Hospitals Case Medical Center and Rainbow Babies and Children's Hospital, Cleveland, Ohio, United States of America; 4 Department of Biomedical Engineering, Case Western Reserve University School of Medicine, University Hospitals Case Medical Center and Rainbow Babies and Children's Hospital, Cleveland, Ohio, United States of America; 5 Department of Physics, University of Würzburg, Würzburg, Germany; University of Alabama-Birmingham, United States of America

## Abstract

**Background:**

Cystic fibrosis (CF) patients would benefit from a safe and effective tool to detect early-stage, regional lung disease to allow for early intervention. Magnetic Resonance Imaging (MRI) is a safe, non-invasive procedure capable of providing quantitative assessments of disease without ionizing radiation. We developed a rapid normalized T1 MRI technique to detect regional lung disease in early-stage CF patients.

**Materials and Methods:**

Conventional multislice, pulmonary T1 relaxation time maps were obtained for 10 adult CF patients with normal spirometry and 5 healthy non-CF control subjects using a rapid Look-Locker MRI acquisition (5 seconds/imaging slice). Each lung absolute T1 map was separated into six regions of interest (ROI) by manually selecting upper, central, and lower lung regions in the left and right lungs. In order to reduce the effects of subject-to-subject variation, normalized T1 maps were calculated by dividing each pixel in the absolute T1 maps by the mean T1 time in the central lung region. The primary outcome was the differences in mean normalized T1 values in the upper lung regions between CF patients with normal spirometry and healthy volunteers.

**Results:**

Normalized T1 (nT1) maps showed visibly reduced subject-to-subject variation in comparison to conventional absolute T1 maps for healthy volunteers. An ROI analysis showed that the variation in the nT1 values in all regions was ≤2% of the mean. The primary outcome, the mean (SD) of the normalized T1 values in the upper right lung regions, was significantly lower in the CF subjects [.914 (.037)] compared to the upper right lung regions of the healthy subjects [.983 (.003)] [difference of .069 (95% confidence interval .032−.105); p = .001). Similar results were seen in the upper left lung region.

**Conclusion:**

Rapid normalized T1 MRI relaxometry obtained in 5 seconds/imaging slice may be used to detect regional early-stage lung disease in CF patients.

## Introduction

Cystic Fibrosis (CF) is the most common life-shortening autosomal recessive disorder in persons of European ancestry[1]. The leading cause of morbidity and mortality in CF patients is progressive respiratory disease. When Dorothy Andersen provided the first comprehensive description of CF in 1938, survival was often measured in days and months[2]. Current expected survival is now 37 years of age[3]. This dramatic improvement in survival has largely occurred due to interventions improving nutrition and treating the airway obstruction, infection, and inflammation in patients with already established CF lung disease[4]. The next logical step to further increasing life expectancy for CF patients is to safely detect and treat CF lung disease at early-stages before irreversible lung damage has occurred[5]. In young children, early-stage CF lung disease can be characterized by significant airway inflammation, infection, and/or obstruction despite no overt clinical symptoms[6]–[9]. Adolescent and adult CF patients with early-stage lung disease are now more common as evidenced by 50% of CF patients in the United States aged ≥18 years having a forced expiratory volume in one second (FEV_1_) value greater than 80% of predicted for a healthy population[3]. Clinical tools such as spirometry, bronchoscopy, and chest imaging are typically used to detect CF lung disease. Unfortunately, these assessments are 1) insensitive to detect early-stage, regional lung disease, 2) invasive, and/or 3) expose patients to potentially injurious ionizing radiation. Therefore a major barrier to improved care for early-stage CF patients is the lack of a safe, non-invasive, and effective test for assessing early, regional lung disease[7].

Magnetic Resonance Imaging (MRI) is a safe, non-invasive procedure capable of providing quantitative assessments of disease without ionizing radiation. MRI techniques, such as hyperpolarized gas imaging as well as proton-based oxygen-enhanced MRI and arterial spin labeling, have been shown to detect CF lung disease[10]–[15]. However, these methods are not widely available, require specialized expensive materials, and/or require multiple lengthy acquisitions resulting in significant respiratory motion artifacts. In addition, no prior studies conducted were focused on detection of early-stage lung disease in CF patients, a timepoint when therapeutics and interventions may be most effective. These practical limitations have prevented clinical adoption of these MRI techniques despite promising initial clinical results.

To address this clinical need, we have developed a normalized T1 MRI (nT1-MRI) technique to quantitatively assess regional CF lung disease. The nT1-MRI technique builds upon previous oxygen-enhanced MRI developments which measure the longitudinal magnetic relaxation time (absolute T1) of lung regions under respired air and oxygen in succession[13]. The T1 magnetic relaxation time generally measures the relative time required for a substance to become magnetized after being placed in a magnetic field. The absolute T1 magnetic relaxation time is a tissue-specific property with adipose tissue and other soft tissue organs having relatively short T1 times (<1000 ms at 1.5T), while blood and other body fluids having a longer T1 relaxation time (>1000 ms at 1.5T). The new nT1-MRI technique described herein requires only one T1 assessment under respired ambient room air and therefore offers the following advantages: 1) rapid image acquisition (5 seconds/imaging slice), 2) minimization of respiratory motion artifacts and elimination of tedious image co-registration, 3) potential to be implemented on virtually any conventional MRI scanner, and 4) no exogenous contrast agents or specialized breathing apparatus are required. Furthermore, since CF lung disease is known to be regional, first appearing in the upper lung regions before the lower regions, and the central lung regions in CF patients with early-stage lung disease typically have no or minimal lung disease[16], we hypothesized that using a region of interest (ROI) analysis, with the “disease-free” central regions acting as a control, the nT1-MRI technique would be sensitive enough to detect early-stage CF lung disease that first appears in the upper lung regions. As such, nT1-MRI may be an ideal diagnostic test for assessing early-stage CF lung disease. Importantly, the normalized T1 technique also improves upon previous studies by reducing subject-to-subject anatomic variation observed in conventional T1 relaxometry assessments and thereby allowing reliable delineation of lung lesions among CF patients.

To determine if the normalized T1 technique may be a potential tool to evaluate regional lung disease in CF patients, we performed an initial cross-sectional study to compare adult CF patients with early-stage lung disease to healthy volunteers. We hypothesized that the nT1-MRI technique would provide a safe and effective tool to detect regional lung abnormalities in early-stage CF patients with “normal” spirometry results.

## Materials and Methods

### Ethics Statement

All patients provided written informed consent and the institutional review board of University Hospitals Case Medical Center approved the study.

### Human Subjects and Spirometry

Adult cystic fibrosis patients were recruited for this initial cross-sectional MRI study from the patient population of the Cleveland Adult Cystic Fibrosis Center at Rainbow Babies and Children's Hospital between June 2012 and February 2013. All MRI scans and spirometry were obtained according to Institutional Review Board approved protocols. Patients were included in the study if they met diagnostic criteria for cystic fibrosis[17] and were aged ≥18 years. Patients were excluded if they had a contraindication to MRI or the patient's respiratory status was not at baseline (i.e. they were experiencing a pulmonary exacerbation). Five healthy adult non-CF subjects with no history of respiratory disease were recruited as controls. Spirometry was obtained for each CF patient and healthy volunteer immediately prior to or immediately following the MRI scan. All spirometry was performed in accordance with American Thoracic Society guidelines and forced expiratory volume in one second (FEV_1_) percent predicted values were calculated using reference equations[18]. For the purposes of this study, we defined early-stage CF lung disease as a CF subject with an FEV_1_≥70% predicted.

### MRI Acquisition

Each subject was scanned in a supine position with a Siemens Espree 1.5T MRI scanner (70 cm bore). Spine array (posterior) and body array (anterior) receiver coils were positioned over each subject's chest to maximize image uniformity. Following initial localizer scans, coronal proton density-weighted HASTE images (Half-Fourier Acquisition Single-shot Turbo spin Echo, respiratory-triggered, TR/TE = 1000/24 ms, slice thickness = 15 mm, 10 slices, FOV = 400 mm×400 mm, partial Fourier factor 5/8, 1 average) were obtained to position the coronal slices for the T1 relaxation assessments. A rapid Look-Locker acquisition was then used to generate T1 recovery data (TR/TE = 1.8 ms/0.84 ms, FOV = 400×400 mm, resolution = 64×128, flip angle = 8°, slice thickness = 15 mm, 40 images following the initial inversion) as described previously[19]. The imaging data were zero-padded to 128×128 isotropic resolution prior to reconstruction and absolute T1 mapping. This acquisition was applied with a 5-second breathhold to obtain T1 recovery data for one imaging slice at a time. The Look-Locker acquisition was then repeated for 8–10 imaging slices to obtain complete lung coverage.

### Absolute and Normalized T1 Calculations and Region of Interest (ROI) Analysis

**Figure 1:**
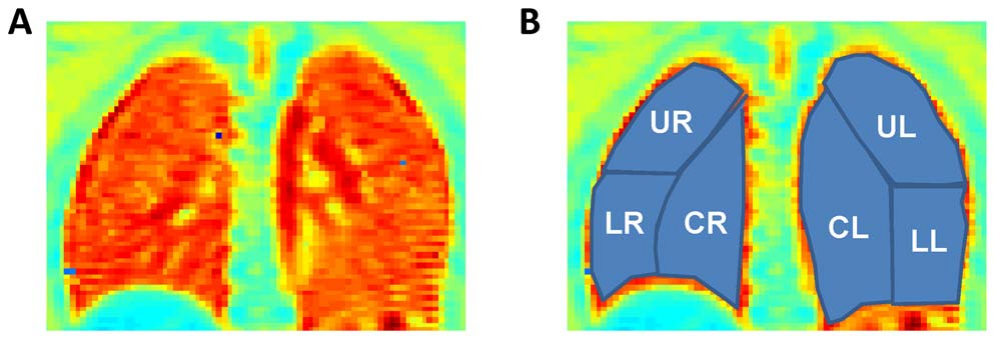
Manual ROI Selection. (a) A representative absolute T1 relaxation time map from a healthy volunteer. (b) The same image with manual ROI's overlaid. (UR = upper right lung region, UL = upper left lung region, CR = central right lung region, CL = central left lung region, LR = lower right lung region, LL = lower left lung region).

Absolute T1 relaxation time maps were calculated online according to established methods[19]. The absolute T1 maps and all MRI images were then exported and processed offline in Matlab (The Mathworks, Natick, MA). A region-of-interest (ROI) analysis was performed to calculate the normalized T1 maps. Six total ROIs were manually selected for each imaging slice as shown in Figure 1. The ROIs were selected with pulmonary and radiological expertise provided by Drs. Dasenbrook and Flask, similar to other previously published CF imaging studies [16], [20]. The central ROI was chosen as the region with the largest pulmonary blood vessels identified by the highest absolute T1 values (dark red regions in Fig. 1a). These regions generally extended from the hilum of the trachea to the base of the lung. The upper and lower ROIs of each lung were chosen from the remaining regions to identify lung disease in the lower and upper halves of each lung. The mean absolute T1 value for the central ROI was then used to calculate the normalized T1 (nT1) value for each image pixel according to Equation 1 below: 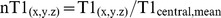
(1)


where nT1_(x,y.z)_ is the normalized T1 value for an individual pixel at spatial location x,y, and z; T1_(x,y.z)_ is the absolute T1 relaxation time (in milliseconds) for an individual pixel at spatial location x,y, and z, and T1_central,mean_ is the mean absolute T1 relaxation time in the central lung region. The central lung region was chosen as a key part of the normalization procedure since CF patients with early-stage lung disease typically do not have lung disease present in the central lung regions. Thus, it is a logical region to serve as a control in order to normalize the measurements and thereby reduce subject-to-subject variation.

The primary outcome reported in the manuscript is the mean normalized T1 value in the 3D upper lung region. Therefore, we used equation 1 above to calculate a pixelwise map representing the normalized T1 value at each spatial location. These maps were then analyzed with an ROI analysis to calculate the mean nT1 value in the upper and lower lung regions for each subject. The ROI analysis was performed over the central 4 imaging slices while excluding imaging slices with heart tissue as well as chest wall signal to avoid confounding in the T1 relaxation data. Overall, the ROIs selected consisted of 10^3^–10^4^ pixels for each subject.

### Statistical Analysis

Mean absolute and normalized T1 relaxation times were plotted as a function of FEV_1_% predicted and forced expiratory flow at 25–75% (FEF(25–75%)) for all CF patients and Pearson correlation coefficients (r) were determined from a least squared error fit to a linear model. The normalized T1 relaxation times and spirometry results from the CF patients and healthy volunteers were compared using two-tailed Student's t-tests. The normalized T1 values from the upper and lower regions were compared among the two groups. Since this was an exploratory study of a novel MRI technique, *a priori* power and sample size estimates were not performed. A two-tailed P-value less than 0.05 was considered statistically significant for all analyses. Analyses were performed using Stata version 10.0 (StataCorp, College Station, Texas).

## Results

**Figure 2:**
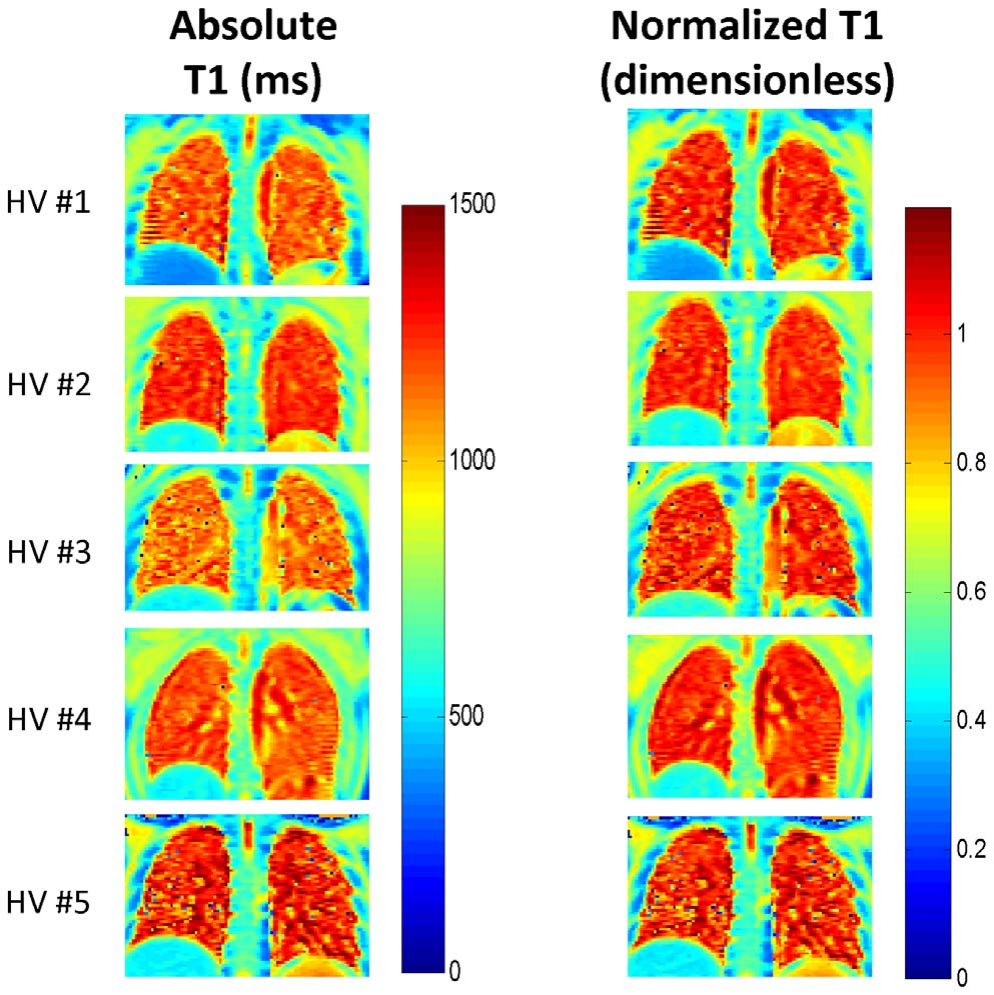
Absolute and Normalized T1 Maps from Healthy Volunteers. Absolute (left column) and normalized (right column) T1 maps from each of five healthy non-CF control subjects. The absolute T1 maps exhibited visible subject-to-subject variation despite the absence of known lung disease. The normalized T1 maps were generated directly from the absolute T1 maps by dividing by the mean central T1 relaxation time for each subject and resulted in reduced subject-to-subject variation for the healthy control subjects. HV: healthy volunteer.

In this cross-sectional study we enrolled 10 CF patients and five healthy volunteers. Subject characteristics are shown in Table 1. CF patients ages ranged from 18–49 years and the mean [standard deviation (SD)] FEV_1_ was 93% predicted (14.5) and ranged from 73% to 123% predicted. The average sweat chloride was 97 (22) mmol per liter and 9/10 subjects had a sweat chloride greater than 60 mmol per liter. Five of the patients had a respiratory culture positive for *Pseudomonas aeruginosa*; four of the five *P. aeruginosa* isolates had a mucoid phenotype. The adult healthy volunteers had no history of respiratory disease and their FEV_1_ values were 87%, 100%, 101%, 104%, and 118% predicted. Absolute T1 maps (left column) and the corresponding normalized T1 maps (right column) from all five healthy control subjects are shown in Figure 2. All of the healthy subjects exhibited relatively uniform T1 relaxation times within each imaging slice as expected (i.e. minimal regional differences in absolute and normalized T1 relaxation times). However, subject-to-subject variation is clearly visible in the absolute T1 maps for the healthy subjects (left column of Fig. 2). In contrast, the normalized T1 maps (right column) exhibited minimal subject-to-subject variation as evidenced by an ROI analysis showing that the variation in the nT1 values in all regions was ≤2% of the mean.

**Table 1:** Baseline Characteristics of the Study Cohort (n = 10).

Category*	Result
Age, years	31 (10); [18–49]
Female, n (%)	4 (40)
FEV_1_% predicted	93 (15); [73–123]
Genotype, n (%)	
ΔF508/ΔF508	6 (60)
ΔF508/Other	4 (40)
Sweat Chloride mmol per liter	97 (22); [46–124]
Respiratory Culture Results, n (%)†	
*Pseudomonas aeruginosa*	5 (50)
Methicillin-sensitive *S. aureus*	5 (50)
Methicillin-resistant *S. aureus*	2 (20)

* Continuous variables presented as mean (standard deviation); [range].

† Subjects could have more than one infection.

**Figure 3:**
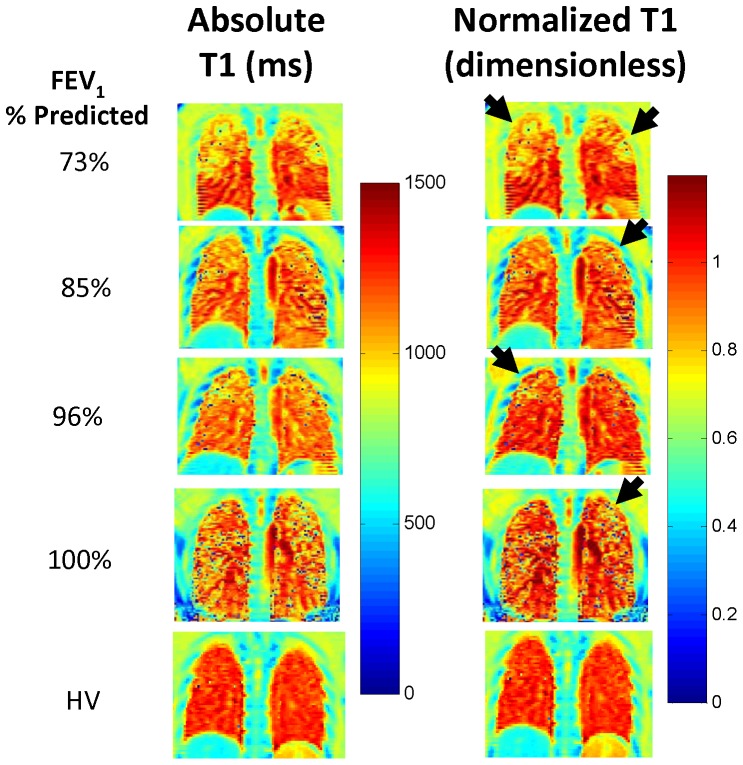
Absolute and Normalized T1 Maps from CF Patients. Representative absolute (left column) and normalized (right column) T1 relaxation time maps from four CF subjects (top four rows, FEV_1_:73%–100% predicted) and one healthy volunteer (HV, bottom row). Regions of decreased normalized T1 indicative of diseased lung are clearly visible in the normalized T1 maps (black arrows). Note also the spatial heterogeneity in the nT1 maps from the CF patients in comparison to the uniform nT1 map of the healthy volunteer (HV).

Representative absolute T1 maps (left column) and normalized T1 maps (right column) from 4 CF patients with a range of pulmonary function (FEV_1_: 73%–100% predicted) are shown in Figure 3. In contrast to the healthy volunteers (HV, bottom row of Fig. 3), CF patients exhibit visible spatial heterogeneity in the nT1 maps. Specifically, all CF patients (including the other 5 CF patients not shown in Fig 3) exhibited regions of decreased nT1 values, particularly in the upper lung regions (black arrows in Fig. 3 right column). Importantly, these observed lung “lesions” are much more conspicuous in the nT1 maps than in the absolute T1 maps demonstrating the importance of the normalization procedure. Further, these lung “lesions” were observed in all CF patients despite “normal” spirometry.

**Figure 4:**
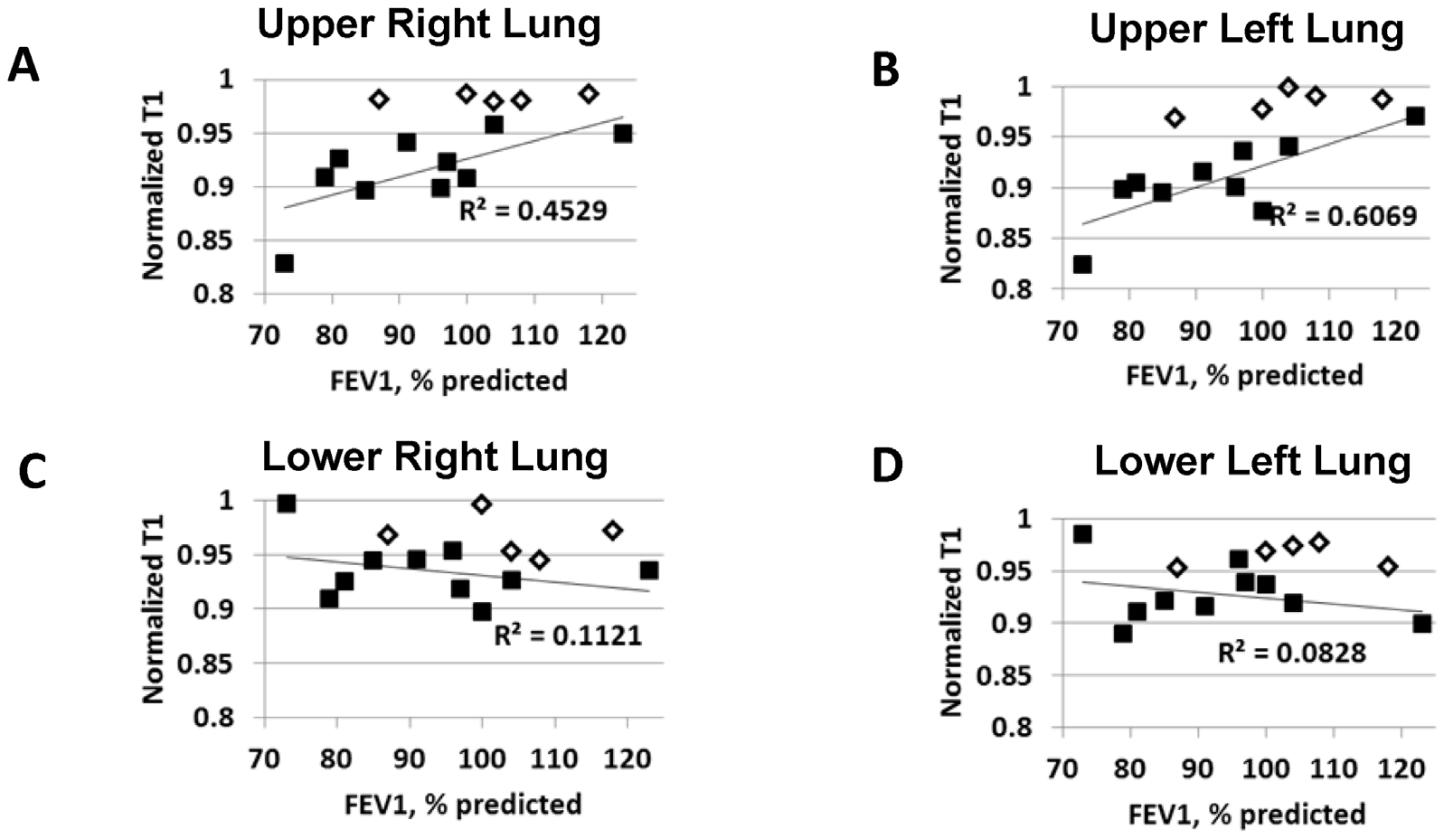
Mean Regional Normalized T1 Values as a Function of FEV_1_% predicted. Mean regional normalized T1 (nT1) values as a function of FEV_1_% predicted for all ten early-stage CF patients (black squares) and 5 healthy volunteers (open diamonds). (a) upper right lung region; (b) upper left lung region; (c) lower right lung region; (d) lower left lung region. Linear regression lines and Pearson Correlation coefficients for the CF patients (controls excluded) are also shown in each plot. The mean nT1 values in the upper left and right lung regions resulted in a significant linear correlation (p<0.05) with FEV_1_% predicted despite the known variation in these spirometric results. As expected, the correlations for the mean normalized T1 assessments in the lower lung regions were not significant (p>0.1). Note also the consistently lower mean nT1 values in the upper right and left lung regions for the CF patients in comparison to healthy volunteers.

In addition to these qualitative results, mean nT1 values were obtained in the upper and lower lung regions of each CF patient and healthy volunteer. The mean nT1 value in the upper right lung region (Fig. 4a), upper left lung region (Fig. 4b), lower right lung region (Fig. 4c), and lower left lung region (Fig. 4d) were plotted as a function of FEV_1_% predicted for all subjects and are shown in Figure 4. Pearson correlation coefficients from a linear regression of the mean nT1 values in each lung region were calculated and are shown in each plot. Pearson correlation coefficients from a linear regression of the mean nT1 values as a function of FEF(25–75%) were also calculated. There was a significant correlation between FEV_1_% predicted and nT1 values in both the upper right and left lung regions (R^2^ = 0.45 and 0.61, respectively; p<.05). There was also a significant correlation between FEF(25–75%) in both the upper right and left lung regions (R^2^ = 0.44 and 0.44, respectively; p<.05). Visual inspection of the plots reveals that the normalized T1 values in the upper lung regions of the CF patients (black squares) are consistently lower than the nT1 values for the healthy volunteers (open diamonds). As expected in CF patients with early-stage lung disease, there was no significant correlation between FEV_1_% predicted or FEF(25–75%) and nT1 values in the lower lung regions.

**Figure 5:**
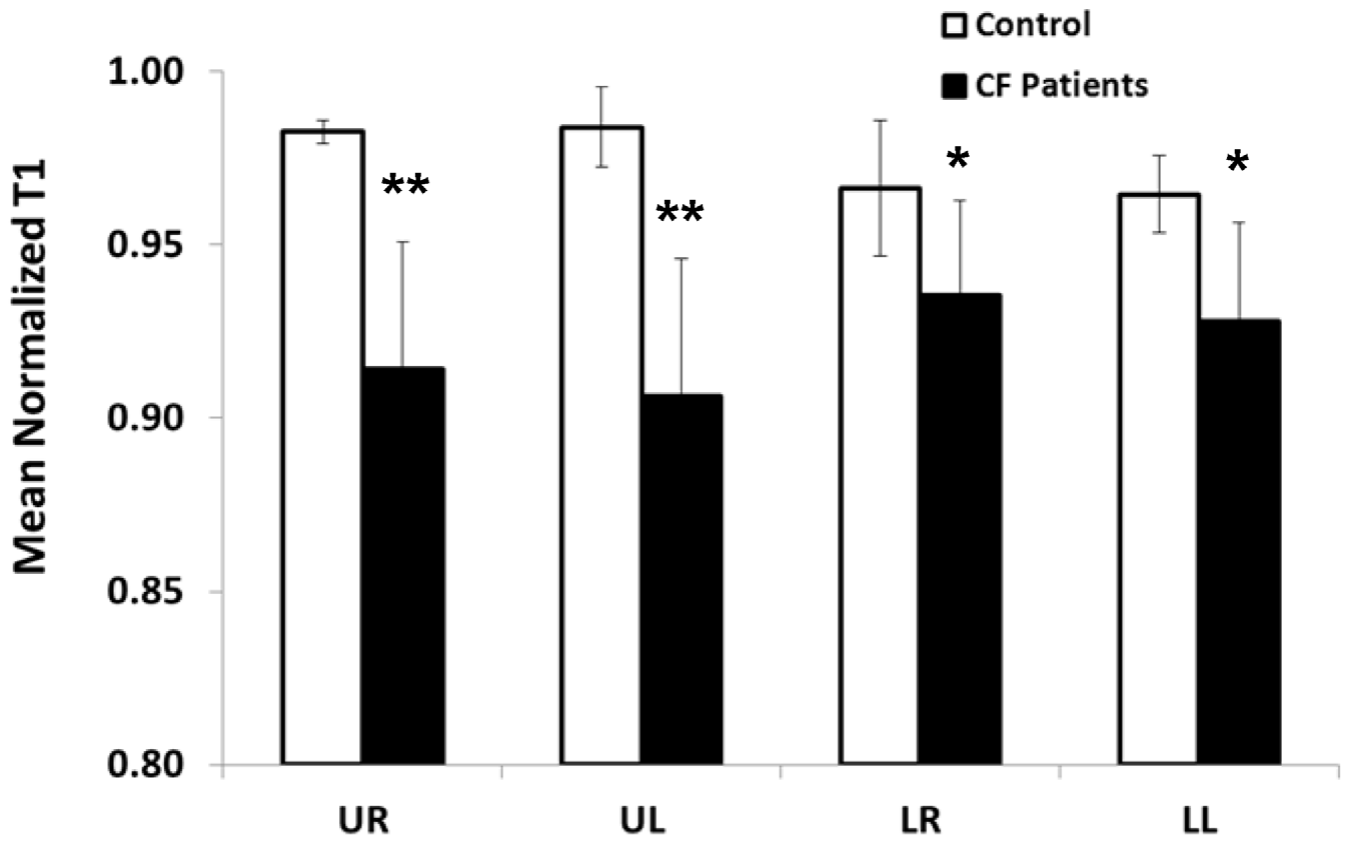
Comparison of mean regional nT1 values from the upper and lower lung regions for the CF patients and healthy volunteers. A significant reduction in mean (SD) nT1 (**p = 0.001) was observed in the upper right (UR) [.914 (.037)] and upper left (UL) [0.906 (.040)] lung regions (black bars) for the CF patients (n = 10) in comparison to the healthy control subjects (n = 5) UR [.983 (.003)] and UL [0.984 (.011)] lung regions (open bars). The mean nT1 in the lower right (LR) and lower left (LL) lung regions was also significantly reduced for the CF patients in comparison to healthy volunteers (*p<0.05). Importantly, these differences were observed despite normal spirometry in both groups.

The primary outcome, a group analysis of the mean normalized T1 values for the CF (n = 10) and healthy control (n = 5) groups, is shown in Figure 5. The grouped data in figure 5 is also presented in scatterplot form in Figure 4. The mean (SD) of the normalized T1 value in the upper right lung of the CF subjects [.914 (.037)] (black bars) was significantly lower than in the upper right lung region of the healthy volunteers [.983 (.003)] (open bars). Thus, the difference between the CF patients and the healthy controls in the upper right lung region was .069 (95% confidence interval (CI) .032 to .105); p = .001). Similarly, the mean nT1 value in the upper left lung region [0.906 (.040)] was significantly lower than the upper left lung region in the healthy subjects [0.984 (.011)] (black bar). Thus, the difference between the CF patients and the healthy controls in the upper left region was .078 (95% confidence interval (CI) .038 to .118); p = .001). The mean nT1 differences between the CF patients and healthy controls in both lower lung regions (.031 and .037 for the lower right and lower left regions, respectively) was as expected, less than observed in the upper lungs regions. However, these differences were still statistically significant due to the low variation in the nT1 methods (p<0.05). Overall, it is important to note that these nT1 differences were detected despite the CF patients having an average FEV_1_ of 93% predicted resulting in no statistical difference in spirometry between early-stage CF patients and healthy volunteers (p>0.05).

## Discussion

In this preliminary cross-sectional study we evaluated if regional lung function can be detected in adult CF patients with early-stage lung disease using nT1-MRI. We found that normalized T1 relaxation times can reliably assess and differentiate lung abnormalities in adult CF patients with early-stage lung disease from healthy volunteers. There are many diagnostic tools that can assess and differentiate later stages of CF lung disease. Therefore, the most interesting finding from this study is that the normalized T1 technique was able to detect early-stage CF lung disease despite “normal” measurements of lung function using the current gold standard, spirometry.

The pathophysiologic processes underlying the observed decreases in lung nT1 values in CF patients shown here, and in absolute T1 relaxation times in previous oxygen-enhanced MRI studies, is currently not established. In CF, dysfunction of the CF transmembrane conductance regulator (CFTR) results in impaired muco-ciliary clearance leading to an environment favorable for infection and inflammation which ultimately destroys the lung[1]. It is hypothesized that early-stage CF lung disease stems from obstruction of the small airways leading to ventilation defects and hypoxic vasoconstriction[21]. Schraml and colleagues studied Arterial Spin Labeling (ASL) MRI, a technique that is very specific for blood flow, in CF patients. Their findings suggest that CF lung disease detectable by MRI is associated with decreased regional pulmonary blood flow[15]. As reduced blood flow would likely result in reduced nT1 values, these ASL-MRI results suggest that the reduced nT1-MRI values we observed in early-stage CF patients (Figures 3–5) are related to reduced pulmonary blood flow. Unfortunately, the ASL MRI techniques require extended acquisition times, multiple image acquisitions, and a much more complicated analysis in comparison to nT1-MRI making the ASL somewhat impractical for routine clinical use. Further preclinical and clinical MRI studies are needed to validate the reduced pulmonary blood flow theory.

The normalized T1 relaxation time assessment builds upon previously reported oxygen-enhanced MRI techniques and provides several major advantages over current diagnostic tests in our effort to develop a viable imaging biomarker for the detection of early-stage lung disease in CF patients of all ages. First, the rapid, normalized T1 assessment provides quantitative MRI data in ∼5 seconds/imaging slice. Patients require 8–10 slices to obtain these results, thus comprehensive 3D nT1-MRI studies could easily be performed in less than 2–3 minutes. Second, in contrast to other techniques such as infant pulmonary function tests or lung clearance index which are not widely available, the nT1 technique is widely generalizable as it can be performed on practically any modern MRI scanner currently available at almost all CF centers around the world. Third, MRI has no ionizing radiation allowing this technique to be used safely to longitudinally assess lung disease in even the youngest of patients (newborns to infants), where the concern of ionizing radiation, especially repeat studies, is heightened. Fourth, this technique does not require the use of inhaled or intravenous contrast agents resulting in a safer and simpler imaging procedure. The nT1 MRI technique eliminates the need to provide supplemental oxygen, as in previously published oxygen-enhanced MRI studies, and intravenous or inhaled MRI contrast agents[10], [13], [22]. Removing the need for oxygen respiration also eliminates tedious image co-registration between the multiple T1 relaxation maps as these images are typically acquired several minutes apart to allow for oxygen/air wash-in. The lack of image co-registration for the nT1-MRI is also a significant improvement over the ASL techniques which require 20-50 image averages to quantify regional lung blood flow. Fifth, our novel normalization process reduces inter-subject variation by using the typically disease-free central lung region as a control to normalize the MRI results in the CF patients with early-stage CF lung disease. One advantage of the previously discussed oxygen-enhanced MRI technique is facilitation of effective subject to subject comparisons using multiple T1 measurements during respiration under room air and at various oxygen concentrations to effectively “normalize” the results. Our results shown here demonstrate that the normalization provided by the nT1-MRI technique also minimizes subject-to-subject variation as evidenced by the low variation in the results for the healthy volunteers. Furthermore, the correlation with FEF(25–75%) suggests detection of small airway disease[21]. FEF(25–75%) is more variable than FEV_1_, making it less optimal as a clinical and research tool[21]. Finally, this functional technique is capable of obtaining regional information as opposed to spirometry which reflects global disease. Taken together, these results suggest that regional, normalized T1 relaxometry may provide a sensitive and clinically effective assessment of early-stage lung disease in CF.

This initial study of nT1 has several limitations. As this was a preliminary study on the functional aspects of MRI in CF patients with early-stage lung disease, structural studies were not performed. Thus we did not correlate the abnormal regional measurements in the upper lobes of the CF patients with structural damage noted on either chest roentegrams or CT. Given that it is known that CF lung disease begins in the upper lobes[16] and the fact that contrast enhanced MRI studies have previously correlated perfusion defects with structural changes[23], the functional changes noted with the nT1-MRI technique most likely represent pathophysiology consistent with early-stage CF lung disease. Future studies will correlate our novel normalization MRI technique with other imaging modalities. Another important limitation of this study is the use of manual ROI selection to perform the regional lung analysis and normalization. The selection of the upper, lower, and central regions of the lungs was primarily guided by the cystic fibrosis and radiologic expertise of the coauthors. Despite this expertise, the ROI selection is inherently subjective. However, each scan generated T1 maps for at least 4 imaging slices. Therefore, each subject's images were used to generate mean T1 values for at least 24 ROIs (4 slices, 3 ROIs/lung, right and left lungs). In this way, the effects of a single erroneous ROI selection on the overall mean T1 relaxation time is somewhat diminished. Overall, the variation observed in the results for the healthy volunteers was much lower for the nT1 technique in comparison to spirometry (≤2% vs 11%). The relatively low variation in the ROI analysis is also confirmed by the consistency of the mean normalized T1 values in the left and right lungs of all subjects as shown in Figures 4 and 5. The central lung region was chosen for normalization of the T1 data primarily because CF lung disease is known to be regional, first appearing in the upper lung regions before the lower regions and the right side before the left.[16], [19] Therefore, the central regions of the lung as shown in Figure 1 represent a region that is not generally expected to contain diseased tissue until the disease has progressed to a much later stage. As a result, for CF patients with early-stage lung disease, the mean absolute T1 values in the central region could be considered to be a “healthy” lung anatomic reference to assess the pathophysiologic changes in the other regions of the lungs. In addition, the central ROIs were predominantly selected because of the large blood vessels easily observed in the absolute T1 maps. Other anatomic regions in other tissues could have been chosen as the reference (e.g. liver). However, normalizing to other tissues would not be expected to correct for the subject-to-subject variation in the lung vasculature and since CF is a systemic disease there may be undetected disease in other tissues.

This technique could potentially benefit not only adults, but newborns and young children with CF. As discussed, a significant number of adolescent and adult CF patients have “normal” lung function as measured by spirometry[3]. Unfortunately, these patients are typically excluded from clinical trials as spirometry lacks the sensitivity to detect changes in CF patients with early-stage lung disease[24]. One primary goal of developing this technique is to eventually apply it to newborns and young children for both clinical and research purposes. It is thought that CF lung disease begins in this young population prior to overt signs and symptoms of lung disease[7], [9], [21], [25]. Thus, tools are needed to detect pre-symptomatic lung disease in these children. Given the advantages of this technique elucidated earlier, it may be a safe and effective diagnostic tool for newborns and young children. This is an exciting time in cystic fibrosis as there are already therapeutics available that treat the basic defect and several more are in the pipeline[26]. It has already been shown that traditional clinical endpoints (i.e. pulmonary exacerbations) may not be sensitive enough to detect early, regional lung disease in newborns and young children and thus new endpoints are needed[5]. Our data, suggests that nT1-MRI is capable of detecting functional changes in the lungs of adult CF patients with early-stage lung disease, thus providing a strong impetus to determine if lung lesions can be detected by nT1-MRI in infant and young children with CF. Taken together, nT1-MRI may be a powerful tool enabling future strategies for the prevention and mitigation of CF lung disease progression. This is vitally important as the reversal of early-stage CF lung disease would result in longer and more fulfilling lives for CF patients.

## Conclusions

In conclusion, the results from this initial study suggest that the normalized T1 MRI technique may provide a reliable and sensitive marker for early-stage lung disease assessments in CF patients. Specifically, the normalized T1 values in the upper lung regions of early-stage CF patients were significantly decreased relative to healthy controls despite “normal” spirometry. This development suggests that normalized T1 relaxometry may provide a safe and effective tool for assessing the extent of lung disease in early-stage CF patients. Further studies are needed to determine the exact mechanism of our findings and to extend the utility of this technique to newborn and young children with CF.
